# Sexual selection and the evolution of obligatory sex

**DOI:** 10.1186/1471-2148-7-245

**Published:** 2007-12-20

**Authors:** Lilach Hadany, Tuvik Beker

**Affiliations:** 1 Department of Biological Sciences, University of Iowa, Iowa City, Iowa 52242, USA

## Abstract

**Background:**

Among the long-standing conundrums of evolutionary theory, obligatory sex is one of the hardest. Current theory suggests multiple factors that might explain the benefits of sex when compared with complete asexuality, but no satisfactory explanation for the prevalence of obligatory sex in the face of facultative sexual reproduction.

**Results and Conclusion:**

We show that when sexual selection is present obligatory sex can evolve and be maintained even against facultative sex, under common scenarios of deleterious mutations and environmental changes.

## Background

Sexual reproduction remains a mystery, and sex as the only mode of reproduction for a species – an even greater mystery. All else being equal, females reproducing asexually have twice the number of their genes transmitted to the next generation, compared with ones that mate with males [1]. Multiple factors, including deleterious mutations [2-4], adaptation events [5-7], parasites [8,9], and combinations of the above [10-12] were suggested to explain the benefits of sex against complete asexuality. However, there is no satisfactory explanation for the prevalence of *obligatory *sex in the face of facultative sexual reproduction. Rather, most of the advantages of sex should still accrue when only a small proportion of the offspring are produced sexually, while the cost of sex would be largely avoided [13-16]. The hardest case for the evolution of obligatory sex occurs in the realistic scenario whereby the reproductive isolation between obligatory and facultative sexuals is incomplete. In such a case the long-term advantages of obligaotry sex are shared with the facultative subpopulation, and the maintenance of obligatory sex depends on its short term benefits. No existing model has identified conditions under which obligatory sex would be favored in this scenario. The maintenance of obligatory sex is especially hard to explain when considering mutant alleles which induce sexual reproduction only when the condition of the individual is poor [17-19]. Such mutants would pay the cost of sex only when needed, and would have the "abandon-ship" advantage: they would be able to break away from unfit genomes and associate themselves to fitter ones, resulting in a strong short term advantage [18,19].

Another factor that occurs in sexual populations but not in asexual ones is sexual selection. In many sexual species, the sex that invests less in the offspring (usually the males) has a higher variation in fitness due to more variable mating success [20]. Such sexual selection can be very strong [21-23], and has been suggested in the past as a factor related to the evolution and maintenance of sex: Darwin [20] observed the effect of sexual selection in enhancing natural selection. Other authors suggested that differential mating success in males would give females fitter daughters [24], and lead to higher average fitness at equilibrium by reducing mutational load [25-28], and improved adaptation to a changing environment [29-31].

Sexual selection can thus enable a sexual population to out-compete purely asexual populations in the long run. In this work we explore the possible role of sexual selection in answering the harder question: how obligatory sex could evolve and be maintained when full or partial mixing occurs between obligatory and facultative sexuals.

**Figure 1:**
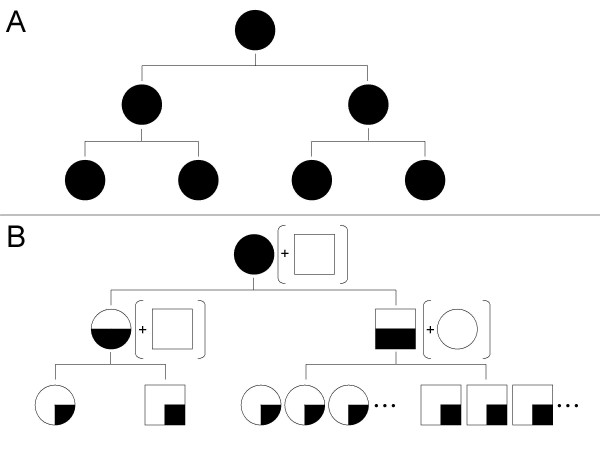
**The effect of sexual selection on the progeny of a fit female**. A female (represented by a circle) that reproduces asexually ensures that all of her genes are transferred to each of her offspring (A). For a highly fit female that reproduces sexually (B), the proportion of her genes in the offspring is diluted by a factor of two each generation (shrinking black areas). However, if males (represented by squares) have differential mating success, then the amount of progeny fathered by a successful male offspring (bottom right) can more than compensate for the recurring twofold cost.

For an intuition about the short-term effect of sexual selection, let us consider the problematic case of the highly fit females. These females would normally have an immediate twofold fitness advantage when reproducing asexually. When considering only first generation descendents, the benefits of sexual selection are restricted to possibly producing better offspring – a considerable advantage to unfit females, but only a limited gain to highly fit ones. But as early as one generation later, sexual selection may offer females a much greater benefit. Indeed, if males are subject to differential mating success, and if fit females tend to have sexually successful offspring, then the males among a fit female's progeny (sons, grandsons, etc.) may easily produce enough offspring to compensate for the 'loss' she experiences in the first generation (Fig. 1). This advantage of sexual selection first appears in the second generation, and can accumulate over later generations. Of course, the advantage to the fittest females is accompanied by a disadvantage to the least fit ones, whose sons would not be as successful due to the deleterious alleles they inherit. However, a gene for sexual reproduction has a different advantage in these less fit females, stemming directly from genetic mixing – it can dissociate from its bad genetic background and potentially proliferate in future generations [18].

## Methods

To study the evolutionary dynamics of a gene coding for obligatory sex, assume we have a locus with two possible alleles. The allele *O *codes for obligatory sex and the allele *F *codes for facultative sex. The population is infinite, and individuals have long haploid genomes exposed to deleterious mutations. The population is fully mixed and no inbreeding occurs. Denote the frequency of newborn individuals with *i *mutations and reproductive strategy *X ∈ *{*O*, *F*} by *p*(*i*, *X*).

The number *d *of new deleterious mutations, added to those inherited by a young individual from its parents, has a Poisson distribution. We neglect mutations in the *O/F *locus. After mutation we get:

$$\tilde{p}(i,X)=\sum_{j=0}^{i}p(j,X)p(d=i-j),\text{ where }d\sim Poisson(U)$$

The probability *ω *of survival of a young individual depends only on the number *i *of mutations it carries: *ω*_*i *_= *e*^-*is*^, where *s *is the strength of natural selection against each deleterious mutation. We assume that individuals carrying *N *or more mutations do not survive. For large enough *N*, the effect of such truncation on the dynamics is negligible. We used *N *= 50 in our analysis. Control cases with *N *= 100 yielded indistinguishable results for the parameter range studied.

The frequencies of the different types after mutation and natural selection are

$$p*(i,X)=\frac{\omega_{i}\tilde{p}(i,X)}{\bar{\omega }},\text{ where }\bar{\omega }=\sum_{i=0}^{N}\omega_{i}\tilde{p}(i,X).$$

An individual of type (*i*, *F*) invests *a *of its resources in asexual reproduction, and 1-*a *in sexual reproduction. An offspring resulting from sexual reproduction has an equal chance of being either a male or a female:

$$\begin{array}{l} p_{females}(i,O)=p_{males}(i,O)=\left(1-C_{sex}\right)p*(i,O)\\ p_{females}(i,F)=p_{males}(i,F)=\left(1-C_{sex}\right)p*(i,F)\cdot \left(1-a\right)\\ p_{asexual}(i,F)=p*(i,F)\cdot a \end{array}$$

Where *P*_*females*_(*i*, *O*) is the proportion of individuals in the population which are females of type (*i*, *O*), and similarly for the other types and genders.*C*_*sex *_is the cost of sex, assumed to be 0.5 if males contribute nothing but their genes to the offspring.

We assume that due to decreased mating success, males are more severely affected by deleterious mutations than females. The mating probability of a male carrying *i *mutations is *φ*_*i *_= *e*^-*isχ*^, where *χ *is the relative strength of sexual selection in comparison with natural selection. Individuals with the *O *and *F *alleles are present in the same population and are allowed to interbreed. The frequencies of different male genotypes among the reproducing males, taking into account sexual selection, are

$${\hat{p}}_{males}(i)=\phi_{i}/\bar{\phi },\text{ where }\bar{\phi }=\sum_{i=0}^{N}\phi_{i}\sum_{X}p_{males}(i,X).$$

Assuming free recombination and a very long genome, the probability of both parents carrying the same mutation is very low. The number of mutations carried by a newborn can thus be approximated by a binomial random variable, taking the success probability to be 0.5 and the number of trials to be the total number of mutations in the two parents. The frequencies of the different types in the next generation are then:

$$\begin{array}{l} p'(i,O)=\sum_{j,k=0}^{N}b(i|k+j)[p_{females}(j,O)\cdot {\hat{p}}_{males}(k,O)+0.5p_{females}(j,O)\cdot {\hat{p}}_{males}(k,F)+\\ +0.5p_{females}(j,F)\cdot {\hat{p}}_{males}(k,O)] \end{array}$$

$$\begin{array}{l} p'(i,F)=\sum_{j,k=0}^{N}b(i|k+j)[p_{females}(j,F)\cdot {\hat{p}}_{males}(k,F)+0.5p_{females}(j,F)\cdot {\hat{p}}_{males}(k,O)+\\ +0.5p_{females}(j,O)\cdot {\hat{p}}_{males}(k,F)]+p_{asexual}(i,F). \end{array}$$

The model can be extended to consider the effect of environmental changes. Let us assume that the environment has one of four states and the suitability of an individual to a particular environment is determined by two loci with two alleles each, where one of the four allele combinations in these loci constitutes a perfect match to the environment. The number j of mismatches between the alleles at these loci and the current environment affects both the viability of the individual and the mating probability in the case of males: The viability of an individual carrying i deleterious mutations and j mismatches with the environment would be *ω*_*i *_= *e*^-*is*-*jt*^, where t is the strength of selection against each mismatch with the environment. The mating success of a male carrying i deleterious mutations and j mismatches would be *φ*_*i *_= *e*^(-*is*-*jt*)*χ*^. In this version of the model we study the frequencies p(E, i, X) of individuals with genotype E in the loci affecting environmental match, i deleterious mutations, and reproductive strategy X (see Additional file 1 for the full form of Equation (5) in this case).

## Results

**Figure 2:**
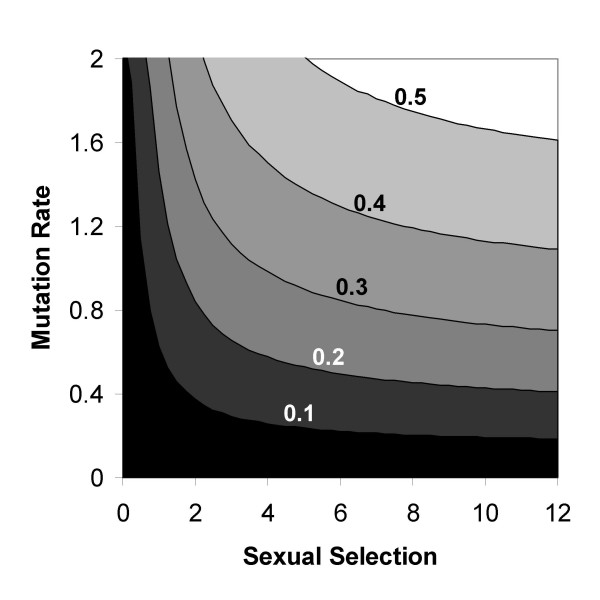
**The quantitative effect of sexual selection on fixation of an allele for obligatory sex**. Plot of the critical genome mutation rate (*U*) above which obligatory sex fixes in a facultative population, as a function of the strength of sexual selection (*χ*). Contour lines with different costs of sex *C*_*sex *_= 0.1,0.2, 0.3, 0.4, and 0.5 are shown. The white area above the *C*_*sex *_= 0.5 contour is the parameter range in which obligatory sex fixes even with a two-fold cost of sex. The plot was generated using *a *= 0.99, *s *= 0.05, and *N *= 50, with free recombination. Fixation of the allele O was defined as reaching a frequency higher than 99.9% within 2500 generations.

Numerically analysing the equations in the case of unchanging environment, we found that sexual selection can favour the short term evolution of obligatory sex on the background of facultative sexuality when genome mutation rate is high enough, even if the cost of sex is significant (Figure 2, different costs of sex and constant environment).

**Figure 3:**
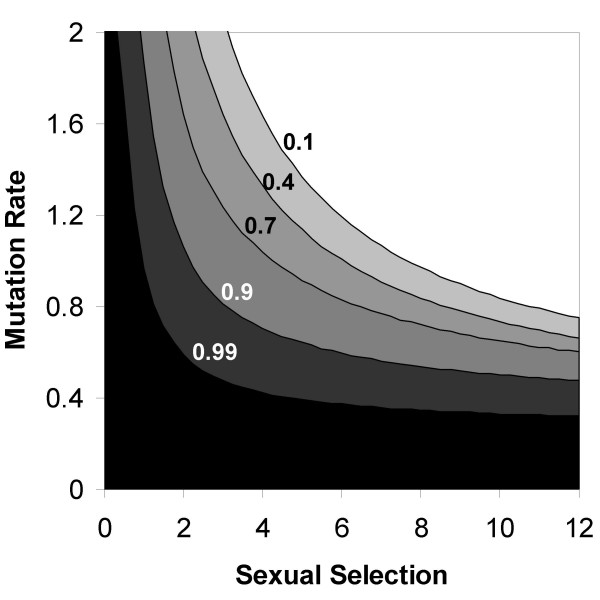
**Conditions for fixation of an allele for obligatory sex in populations with different levels of facultative sex**. The contours represent the critical mutation rate above which obligatory sex fixes. Contour lines are shown for different frequencies of sexual reproduction among the facultative sexuals: *a *= 0.1,0.4, 0.7, 0.9, and 0.99. The white area above the *a *= 0.1 contour is the parameter range in which obligatory sex fixes even in a population where only 0.1 of the offspring are produced asexually. The plot was generated using *C*_*sex *_= 0.2, *s *= 0.05, and *N *= 50, with free recombination. Fixation of the allele O was defined as reaching a frequency higher than 99.9% within 2500 generations.

The effect was sensitive to the frequency of asexuality, *a*, among the facultative sexuals. Higher values of asexuality among the facultatives allow obligatory sex to evolve more easily (see Figure 3, different contours representing different values of *a*). However, our model assumes free recombination, resulting in a dramatically increased effect for facultative sexuals that rarely reproduce sexually. Furthermore, infinite population means that any amount of sexual reproduction is sufficient to produce all possible genotype combinations. The critical values of *a *in our analysis are therefore likely to greatly overestimate its critical values in finite populations, and in particular in populations where the rate of recombination is limited.

**Figure 4:**
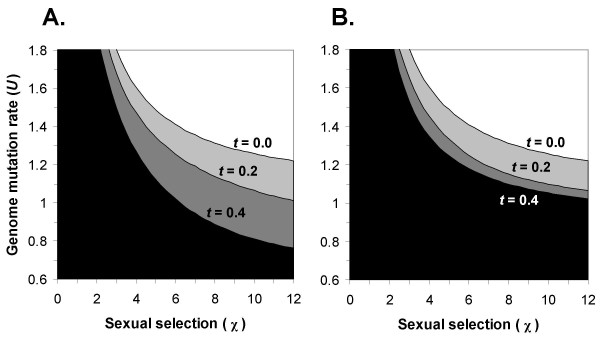
**Conditions for fixation of an allele for obligatory sex under environmental changes**. The contours represent the critical mutation rate above which obligatory sex fixes. Contour lines with *t *= 0,0.2 and 0.4 are shown. The white area above the *t *= 0 contour is the parameter range in which obligatory sex fixes even in a constant environment. The plot was generated using *C*_*sex *_= 0.5, *a *= 0.99, *s *= 0.05, and *N *= 50, with free recombination. Environmental changes occurred every 10 generations (A) or every 25 generations (B). Fixation of the allele O was defined as reaching a frequency higher than 99.9% within 2500 generations and retaining it for four consecutive cycles of environmental change.

Using the extended model (see Additional file 1) we found that environmental changes can significantly expand the parameter range under which obligatory sex evolves, compared with deleterious mutations acting alone. The effect of environmental changes varies in magnitude, depending both on the selection *t *on adaptation loci (compare the different contours in Figure 4A) and on the frequency of environmental changes (compare Figure 4A with 4B). When environmental changes occur less frequently, the benefit gained from faster adaptation diminishes, and the advantage to the obligatory sexual individuals is weakened.

## Discussion

The advantages of sex that result from sexual selection are inherently different from those resulting from recombination or segregation. The latter two can increase the efficiency of natural selection indirectly, by affecting the distribution of different genotypes in the population [32,33]. But they do not offer a successful female any benefit from reproducing sexually herself. Sexual selection, on the other hand, results in a direct advantage to the fittest genotypes, if and only if they reproduce sexually.

The first and most direct prediction of this model is that obligatory sex would be more common among organisms with highly differential mating success. In this context, strong sexual selection is not limited to cases of extreme handicaps. It also applies to any case of high choosiness of females or intense competition between males over territory or hierarchy. This prediction is consistent with the general observation that obligatory sex is more common, whereas sexual selection is often stronger, among animals than among plants: Plants do exert intense selection on pollen, which is indicative of the quality of gametes as well as the ability of the mate to produce gametes that are effectively dispersed [34,35]. Nevertheless, they are inherently limited in their ability to compare the overall quality of potential mates [36]. Second, similar to models for the advantage of sex vs. asexuality, obligatory sex is expected to be more common among organisms experiencing higher genome mutation rates [2,3]. The genome-wide rate of deleterious mutations in natural populations is still largely controversial [37-40]. Recent direct estimates suggest that the rates might be higher than previously expected, and may reach over 1 mutation per genome per generation even in short-lived organisms such as *Drosophila melanogaster *[41]. Third, our model predicts that obligatory sex would be more common under environmental changes, and in particular under environmental changes with strong effect on the fitness. Parasites may constitute one possible source of such changes [9,42,43], and explicit models of obligatory sex under red queen scenarios could provide further insight into their effect.

In our model, male mating success is affected both by deleterious mutations and by environmental changes. While a female cannot easily determine what makes a potential mate maladapted, she often has indications of its overall success in competition over territory, for example [21]. A more complicated situation occurs when male display (e.g. song or colours) does not involve direct competition. However, it has been shown by Lorch *et al.*[29] that male display is likely to evolve to be condition-dependent, thus affected by both deleterious mutations and environmental changes. Our assumption is therefore that male mating success is correlated with its overall adaptation to its current environment, which does not require direct choice for specific 'good genes'.

One limitation of our model is the assumption of free recombination. Free recombination results in a significantly increased effect of sex, in terms of breaking genetic associations. The effect is especially dramatic for facultative sexuals, particularly ones that reproduce sexually with a low probability. Obligatory sex is therefore likely to be favoured under wider conditions in populations where the level of recombination is limited. Further research using detailed genetic models that allow low levels of recombination and explicit chromosome structure would enable more accurate quantitative analysis.

Our model assumes that overall fitness is positively correlated with mating success. Such associations have been documented in various organisms [44-50], but do not apply genome-wide, and there are even conflicts between genes that are advantageous for females and ones that benefit males [51]. However, the correlation between the sexual success of a parent and the success of its offspring does not have to be entirely genetic. For example, it can be mediated through higher maternal investment in the offspring of fitter mothers or more attractive fathers [52,53]. Under differential maternal investment and male mating success, sex ratio is expected to be male-biased in fitter females [54], as was empirically demonstrated in several cases [55,56]. We expect obligatory sex to be favoured under wider conditions when sex ratio is thus biased. Finally, the effect of sexual selection on the evolution of obligatory sex is not limited to the specific model of selection presented here. Very similar results are obtained if each female selects the best of *n *male candidates as a mate.

## Conclusion

The fitness benefits of genetic mixing are predicted by many evolutionary models, and some degree of genetic mixing indeed occurs in most organisms, including bacteria and viruses. But for sexual reproduction to become the *sole *mode of reproduction for so many organisms, an additional factor seems to be required. Sexual selection presents one such factor, offering both short-term and long-term advantages to sexually reproducing individuals. As such, it may have played a key role in the evolution of obligatory sex.

## Authors' contributions

Both authors contributed equally to the formulation of the model, the analysis, and the writing of the manuscript.

## Supplementary Material

Additional File 1 A detailed model for the case of periodic environmental changes Click here for file
