# Supplementary material for: Sexual selection and the evolution of obligatory sex
Source: BMC Evol Biol. 2007 Dec 20;7:245. doi: 10.1186/1471-2148-7-245 (PMC2248195; doi:10.1186/1471-2148-7-245)
Supplement: Additional File 1 — A detailed model for the case of periodic environmental changes [file 1471-2148-7-245-S1.PDF]

Sexual selection and the evolution of obligatory sex

Lilach Hadany & Tuvik Beker

Model Details

When modelling the evolution of obligatory sex with both deleterious mutations and environmental changes, we added two loci with two alleles each (A/a and B/b) determining the suitability of the individual to the changing environment. We thus divided each class of genotypes carrying i deleterious mutations with reproduction strategy X into 4 types, according to their genotype at the 2 'adaptation loci', with the following frequencies: $p(A,B,i,X)$, $p(a,B,i,X)$, $p(A,b,i,X)$, and $p(a,b,i,X)$. Equations 1a and 1b are then substituted by 8 equations of the following form:

$$p(A,B,i,O)=\sum_{j,k=0}^{N} b(i\,|\,j+k)$$

$$\left\{\begin{aligned}
& p_{females}(A,B,j,O)\left[\begin{aligned}&\hat{p}_{males}(A,B,k,O)+\frac{1}{2}\hat{p}_{males}(a,B,k,O)\\ &+\frac{1}{2}\hat{p}_{males}(a,B,k,O)+\frac{1}{2}\hat{p}_{males}(A,b,k,O)+\frac{1}{4}p_{males}(a,b,k,O)\end{aligned}\right]\\
& +\hat{p}_{males}(A,B,k,O)\left[\frac{1}{2}p_{females}(a,B,j,O)+\frac{1}{2}p_{females}(a,B,j,O)+\frac{1}{2}p_{females}(A,b,j,O)+\frac{1}{4}p_{females}(a,b,j,O)\right]\\
& +\frac{1}{4}p_{females}(A,b,j,O)\hat{p}_{males}(a,B,k,O)+\frac{1}{4}p_{females}(a,B,j,O)\hat{p}_{males}(A,b,k,O)\\
& +\frac{1}{2}p_{females}(A,B,j,O)\left[\begin{aligned}&\hat{p}_{males}(A,B,k,F)+\frac{1}{2}\hat{p}_{males}(a,B,k,F)\\ &+\frac{1}{2}\hat{p}_{males}(a,B,k,F)+\frac{1}{2}\hat{p}_{males}(A,b,k,F)+\frac{1}{4}p_{males}(a,b,k,F)\end{aligned}\right]\\
& +\frac{1}{2}\hat{p}_{males}(A,B,k,F)\left[\frac{1}{2}p_{females}(a,B,j,O)+\frac{1}{2}p_{females}(a,B,j,O)+\frac{1}{2}p_{females}(A,b,j,O)+\frac{1}{4}p_{females}(a,b,j,O)\right]\\
& +\frac{1}{8}p_{females}(A,b,j,O)\hat{p}_{males}(a,B,k,F)+\frac{1}{8}p_{females}(a,B,j,O)\hat{p}_{males}(A,b,k,F)\\
& +\frac{1}{2}p_{females}(A,B,j,F)\left[\begin{aligned}&\hat{p}_{males}(A,B,k,O)+\frac{1}{2}\hat{p}_{males}(a,B,k,O)\\ &+\frac{1}{2}\hat{p}_{males}(a,B,k,O)+\frac{1}{2}\hat{p}_{males}(A,b,k,O)+\frac{1}{4}\hat{p}_{males}(a,b,k,O)\end{aligned}\right]\\
& +\frac{1}{2}\hat{p}_{males}(A,B,k,O)\left[\frac{1}{2}p_{females}(a,B,j,F)+\frac{1}{2}p_{females}(a,B,j,F)+\frac{1}{2}p_{females}(A,b,j,F)+\frac{1}{4}p_{females}(a,b,j,F)\right]\\
& +\frac{1}{8}p_{females}(A,b,j,F)\hat{p}_{males}(a,B,k,O)+\frac{1}{8}p_{females}(a,B,j,F)\hat{p}_{males}(A,b,k,O)
\end{aligned}\right\}$$

$$p(A,B,i,F)=\sum_{j,k=0}^{N} b(i\mid j+k)$$

$$\left\{\begin{array}{l}
p_{females}(A,B,j,F)\left[\begin{array}{l}\hat{p}_{males}(A,B,k,F)+\frac{1}{2}\hat{p}_{males}(a,B,k,F)\\ +\frac{1}{2}\hat{p}_{males}(a,B,k,F)+\frac{1}{2}\hat{p}_{males}(A,b,k,F)+\frac{1}{4}\hat{p}_{males}(a,b,k,F)\end{array}\right]\\
+\hat{p}_{males}(A,B,k,F)\left[\frac{1}{2}p_{females}(a,B,j,F)+\frac{1}{2}p_{females}(a,B,j,F)+\frac{1}{2}p_{females}(A,b,j,F)+\frac{1}{4}p_{females}(a,b,j,F)\right]\\
+\frac{1}{4}p_{females}(A,b,j,F)\hat{p}_{males}(a,B,k,F)+\frac{1}{4}p_{females}(a,B,j,F)\hat{p}_{males}(A,b,k,F)\\
+\frac{1}{2}p_{females}(A,B,j,O)\left[\begin{array}{l}\hat{p}_{males}(A,B,k,F)+\frac{1}{2}\hat{p}_{males}(a,B,k,F)\\ +\frac{1}{2}\hat{p}_{males}(a,B,k,F)+\frac{1}{2}\hat{p}_{males}(A,b,k,F)+\frac{1}{4}\hat{p}_{males}(a,b,k,F)\end{array}\right]\\
+\frac{1}{2}\hat{p}_{males}(A,B,k,F)\left[\frac{1}{2}p_{females}(a,B,j,O)+\frac{1}{2}p_{females}(a,B,j,O)+\frac{1}{2}p_{females}(A,b,j,O)+\frac{1}{4}p_{females}(a,b,j,O)\right]\\
+\frac{1}{8}p_{females}(A,b,j,O)\hat{p}_{males}(a,B,k,F)+\frac{1}{8}p_{females}(a,B,j,O)\hat{p}_{males}(A,b,k,F)\\
+\frac{1}{2}p_{females}(A,B,j,F)\left[\begin{array}{l}\hat{p}_{males}(A,B,k,O)+\frac{1}{2}\hat{p}_{males}(a,B,k,O)\\ +\frac{1}{2}\hat{p}_{males}(a,B,k,O)+\frac{1}{2}\hat{p}_{males}(A,b,k,O)+\frac{1}{4}\hat{p}_{males}(a,b,k,O)\end{array}\right]\\
+\frac{1}{2}\hat{p}_{males}(A,B,k,O)\left[\frac{1}{2}p_{females}(a,B,j,F)+\frac{1}{2}p_{females}(a,B,j,F)+\frac{1}{2}p_{females}(A,b,j,F)+\frac{1}{4}p_{females}(a,b,j,F)\right]\\
+\frac{1}{8}p_{females}(A,b,j,F)\hat{p}_{males}(a,B,k,O)+\frac{1}{8}p_{females}(a,B,j,F)\hat{p}_{males}(A,b,k,O)
\end{array}\right\}$$

$$+\,p_{asexual}(A,B,i,F)$$

The rest of the equations can be derived similarly (available upon request).
